# Malaria Parasitemia and Severe Health Complications in Children Under Five Years of Age in Nigeria: A Study Using the Demographic and Health Survey (DHS) Malaria Indicator Survey (MIS) 2021

**DOI:** 10.7759/cureus.58907

**Published:** 2024-04-24

**Authors:** Olajide J Olagunju, Onyeka C Ekwebene, Olayinka E Olagunju, Olagoke Osanyinlusi, Oladayo A Oyebanji, Ben Egbo

**Affiliations:** 1 Infectious Disease, Case Western Reserve University School of Medicine, Cleveland, USA; 2 Biostatistics and Epidemiology, East Tennessee State University, Johnson City, USA; 3 Zoology, Obafemi Awolowo University, Ile-Ife, NGA; 4 Pulmonology, Washington University School of Medicine in St. Louis, St. Louis, USA; 5 Radiology, Potiskum Medical Centre, Potiskum, NGA

**Keywords:** malaria, climate, severe health complications, public health challenge, parasitemia

## Abstract

Background: In Nigeria, 97% of the population is at risk of contracting malaria. It is transmitted by female *Anopheles *mosquitoes carrying the *Plasmodium *parasite and can be lethal. An estimated 55 million illnesses and 80,000 deaths per year result from it. Children under five are more likely to contract malaria. Efforts to control malaria in Nigeria include indoor residual spraying, insecticide-treated bed nets, and quick detection and treatment of confirmed cases with effective antimalarial medications. These attempts have been impeded by limited healthcare access, poor financing, and drug-resistant parasites. Thus, the study of the relationship between malaria complications and housing for children under five is essential.

Methods: The Demographic and Health Survey (DHS) Malaria Indicator Survey (MIS) 2021, a nationally representative data set from developing countries on population and health, was used for this study. A sample size of 13,727 was employed (n=13,727). Logistic regression analyses were conducted to test the association between the type of place of residence and malaria complications (outcome).

Results: Overall, 4.2% (n=570, weight HV005) of participants in the sample reported malaria complications. The results of the logistic regression revealed that children residing in urban settlements (aOR 0.37, 95% CI 0.37-0.37, p-value <0.001), children from the poorest class families (aOR 11.63, 95% CI 1.62-1.63, p-value 0.004), children from poorer class families (aOR 7.56, 95% CI 7.55-7.57, p-value <0.001), children from middle-class families (aOR 4.05, 95% CI 4.03-9.06, p-value <0.001), children from richer class families (aOR 1.22, 95% CI 2.21-2.23, p-value <0.001), children of mothers with primary education (aOR 0.42, 95% CI 2.32-4.112, p-value 0.001), children of mothers with secondary education (aOR 0.24, 95% CI 3.21-3.22, p-value <0.001), children of mothers with higher education (aOR 0.08, 95% CI 0.72-0.80, p-value <0.001), and children of the female gender (aOR 0.65, 95% CI 0.65-0.66, p-value <0.001) are all associated with severe malaria complications.

Conclusions: In conclusion, the study examined malaria complications in Nigerian children under five by residency. The findings imply that rural children are more likely to have serious malaria complications than urban children. This emphasizes the necessity for targeted malaria therapies in rural areas with limited healthcare access.

## Introduction

Malaria has been a major public health challenge for decades. It is a life-threatening disease, transmitted through the bites of infected female Anopheles mosquitoes [1-3]. Infected individuals usually have an acute febrile illness which, if not immediately treated, may progress to severe illness within 48 hours, with a high fever associated with chills and rigor, severe headache, vomiting, and body pains [2,3]. However, malaria and its sequelae can be adequately prevented at different levels or effectively managed by appropriate health workers.

About 247 million cases of malaria and an estimated 619 deaths were reported by the World Health Organization (WHO) as of 2021 [4]. The highest burden of malaria is noticeably reported in sub-Saharan Africa, where about 95% of the global burden was diagnosed, and about 96% of the global deaths were recorded by WHO. Unfortunately, about 80% of all malaria deaths in this region were recorded in children under the age of five years. The 2020 World Malaria Report documented the highest number of malaria cases as of the year 2018; about 27% of the global cases of malaria were reported in Nigeria. This accounted for about 23% of the global deaths caused by malaria [5-7].

Malaria remains a leading cause of mortality in Nigeria, with a high rate of mortality across all age groups. It has been observed to have exceptionally higher morbidity and mortality among children under five years of age, pregnant women, travelers from locations with no malaria, and the elderly [8]. It is therefore important to study the risk of severe health complications like extreme weakness, rapid or difficult breathing, seizure, and jaundice that could be complications of diagnosed cases of malaria in Nigeria. Deaths from malaria have been described as directly linked to one or more of these health complications [9,10].

Nigeria is a tropical country with a favorable climate for mosquitoes. The female *Anopheles* mosquito is known for transmitting *Plasmodium *parasites, including *Plasmodium falciparum*, *Plasmodium ovale*, *Plasmodium vivax*, and *Plasmodium malariae*. Severe health complications and possible death from malaria are due to *Plasmodium falciparum *infestation [11]. These complications develop rapidly with a highly significant possibility of progression to death within a few days if not properly managed. Also, two or more of these severe health complications can exist together or evolve in quick succession within a few hours or days [11].

Based on the recommendation of the WHO, Nigeria has adopted the 3T strategy (test, treat, and track), enabling proper diagnosis of suspected cases of malaria with Rapid Diagnostic Test (RDT) kits or microscopy and prompt treatment with artemisinin-based combination therapy (ACT), if the result is positive and documented [12-14]. However, available statistics do not indicate a reduction in the prevalence of malaria and its sequelae.

Multiple studies have examined malaria and severe malaria, especially in vulnerable populations like children under five years of age, pregnant women, travelers from locations with no malaria, and the elderly [1,3,5]. While some malaria infestations are acute and uncomplicated, some are severe with complications. However, none of these studies have examined the risk of severe health complications in malaria infestation [1-3,5]. The outcomes of malaria depend largely on these health complications.

This study focuses on the risk of developing severe health complications in every RDT-positive result based on the type or place of residence. It further explains why some malaria infestations lead to severe morbidity and/or mortality while others do not. This will enable frontline health workers in Nigeria to identify cases of malaria that need to be attended to with a high level of urgency.

The objectives of this study are to determine the risk of severe health complications for every RDT-positive case of malaria, to determine if there is a difference in the risk of malaria complications based on the type of place of residence in children below the age of five years in Nigeria, and to effectively prioritize the management of diagnosed cases of malaria in Nigeria.

## Materials and methods

This cross-sectional study utilized data from the Demographic and Health Survey (DHS) Malaria Indicator Survey (MIS) 2021. The DHS provides nationally representative data in developing countries on population and health. A total of 13,727 children under five years of age were used for this study (n = 13,727, weight = HV005). The independent variable is the type or place of residence, while the main dependent variable is malaria complication, which is a combination of all the complications (extreme weakness, difficulty with breathing, seizure, and jaundice) being examined. The composite variable, with two levels of responses (Present and Absent), has a total observation of 13,727, while 17 children reported having malaria complications, and 13,710 children did not report malaria complications.

The inclusion criteria for the study participants focus on age, encompassing children under the age of five who have been diagnosed with malaria. Children diagnosed with malaria using the RDT kit, and those who have experienced severe health consequences from malaria such as acute weakness, rapid or difficult breathing, seizure, and jaundice or yellow skin, are included. Additionally, children residing in urban or rural settings and diagnosed with malaria are considered. The exclusion criteria involve children over the age of five, whether diagnosed with malaria or not, and those diagnosed using diagnostic modalities other than the RDT kit.

This population represents a vulnerable group susceptible to malaria infestation. The study explores the risk of severe malaria complications based on the type of residence in diagnosed cases of malaria. A total of 570 children were included in the logistic regression analysis. Descriptive statistics were employed to understand the characteristics of the study population. Simple logistic regressions were performed to ascertain the independent association of the explanatory variable and covariates with the outcome. The backward selection method was then utilized to select variables for the multivariate logistic regression, aiming to determine the combined association between malaria complications and the type of place of residence. Variables that were significant in the simple logistic regression were chosen for the multivariate analysis to test the combined association. The Statistical Analysis System (SAS) version 9.4 for Windows (SAS Institute Inc., Cary, North Carolina) was used for the statistical analysis.

## Results

The frequency distribution of the study revealed that, of the total weighted sample size, 66.78% lived in rural areas and did not have malaria complications while 0.10% lived in rural areas and had malaria complications. On the other hand, 33.07% lived in urban areas and did not have malaria complications while 0.05% lived in urban areas and had malaria complications (Table 1).

**Table 1:** Frequency Distribution

Variables		Unweighted		Weighted	
Residence	Malaria Complication	N = 13,727	%	N = 1.3727E10	%
Rural	Absent	9024	65.74	9167181985	66.78
	Present	13	0.09	13,325,446	0.10
Urban	Absent	4686	34.14	453,971,445	33.07
	Present	4	0.03	6,921,055	0.05
Wealth index					
Poorest	Absent	2081	15.16	2,215,660,972	16.14
	Present	3	0.02	3,167,279	0.02
Poorer	Absent	2441	15.60	2,362,341,401	17.21
	Present	3	0.02	2,968,171	0.02
Middle	Absent	2699	19.66	2,699,240,298	19.06
	Present	6	0.04	8,042,658	0.06
Richer	Absent	3189	23.23	3,012,106,967	21.94
	Present	4	0.03	5,593,141	0.04
Richest	Absent	3600	26.23	3,417,403,792	24.90
	Present	1	0.01	47,252	0.01
Mother’s education					
No education	Absent	2368	38.96	2,631,242,765	41.53
	Present	9	0.15	12,307,672	0.19
Primary	Absent	880	14.47	938,705,691	14.82
	Present	2	0.03	2,435,070	0.38
Secondary	Absent	2062	33.93	2,003,070,264	31.61
	Present	5	0.08	4,448,920	0.07
Higher	Absent	752	12.37	743,545,355	11.74
	Present	0	0.00	0	0.00
Gender					
Female	Absent	3393	48.60	3,490,233,217	48.35
	Present	8	0.12	9,794,821	0.14
Male	Absent	3572	51.29	3,707,027,250	51.36
	Present	9	0.13	10,451,680	0.15

Considering the wealth index, 16.14% of children from the poorest families did not have malaria complications, while 0.02% had malaria complications; 17.21% of children from poorer families did not have malaria complications, while 0.02% had malaria complications; 19.66% of children from middle-class families did not have malaria complications, while 0.06% had malaria complications; 21.94% of children from richer families did not have malaria complications, while 0.04% had malaria complications; and 24.90% of children from the richest families did not have malaria complications, while 0.01% had malaria complications.

Considering the mother’s level of education, 41.54% were children of mothers with no education who did not have malaria complications, while 0.19% had malaria complications. 14.82% were children of mothers with primary education who did not have malaria complications, while 0.38% had malaria complications. 31.61% were children of mothers with secondary education who did not have malaria complications, while 0.07% had malaria complications. 11.74% were children of mothers with higher education who did not have malaria complications, and none had malaria complications.

Considering gender, 48.35% were female children who did not have malaria complications, while 0.14% had malaria complications. 51.36% were male children who did not have malaria complications, while 0.15% had malaria complications.

The result of the logistic regression analysis revealed that children residing in urban settlements were 63% less likely to develop malaria complications (aOR 0.37, 95% CI 0.37-0.37, p-value <0.001) compared with children residing in rural settlements. Children from the poorest class families had 11.6 times the odds of developing malaria complications compared with children of the richest class families (aOR 11.63, 95% CI 1.62-1.63, p-value 0.004), poorer class children had 7.6 times the odds of developing malaria complications compared with children of the richest class (aOR 7.56, 95% CI 7.55-7.57, p-value <0.001). Middle-class children had 4.1 times the odds of developing malaria complications compared with children of the richest class (aOR 4.05, 95% CI 4.02-9.06, p-value <0.001), while richer class children had 1.2 times the odds of developing malaria complications compared with children of the richest class families (aOR 1.22, 95% CI 2.21-2.23, p-value <0.001) (Table 2). Children of mothers with primary education were 58% less likely to develop malaria complications compared with children of mothers with no education (aOR 0.42, 95% CI 2.32-4.112, p-value 0.001), children of mothers with secondary education were 76% less likely to develop malaria complications compared with children of mothers with no education (aOR 0.24, 95% CI 3.21-3.22, p-value <0.001), while children of mothers with higher education were 92% less likely to experience malaria complications (aOR 0.08, 95% CI 0.72-0.80, p-value <0.001). Female children were 35% less likely to develop malaria complications compared with male children (aOR 0.65, 95% CI 0.65-0.66, p-value <0.001) (Table 2).

**Table 2:** Logistic Regression

	Unadjusted			Adjusted		
Variable	Point Estimate	95% Confidence Limits	P-Value	Point Estimate	95% Confidence Limits	P-Value
Residence (reference: rural)						
Urban vs rural	0.329	0.328–0.329	<0.001	0.373	0.373–0.374	<0.001
Mother’s education level (reference: no education)						
Primary vs no education	0.58	2.138–5.822	0.001	0.42	2.321–4.112	0.001
Secondary vs no education	0.31	3.477–3.481	<0.001	0.24	3.206–3.215	<0.001
Higher vs no education	0.16	0.115–1.119	<0.001	0.08	0.718–0.800	<0.001
Wealth index (reference is richest)						
Poorest vs richest	10.402	1.400–1.405	<0.001	11.630	1.621–1.633	0.004
Poorer vs richest	7.393	5.393–5.383	<0.001	7.563	7.551–7.575	<0.001
Middle class vs richest	4.678	4.671–4.686	<0.001	4.046	4.029–9.063	<0.001
Richer vs richest	1.655	0.653–0.651	<0.001	1.220	2.212–2.228	<0.001
Gender (reference: male)						
Female vs male	0.760	0.759–0.761	<0.001	0.648	0.647–0.659	<0.001

## Discussion

Malaria remains a major public health concern in Nigeria, particularly among children under five years old. This study aimed to explore the relationship between the type of place of residence and malaria complications in children under five in Nigeria. We found that the type of place of residence, wealth index, mother's level of education, and gender are all significantly associated with the risk of malaria complications in this age group. These findings have important implications for malaria control and prevention efforts in Nigeria. 

The key finding of the study was that children living in rural areas were at a higher risk of malaria complications compared to those living in urban areas. Children under five years of age residing in rural settlements in Nigeria are at a higher risk of malaria complications due to several factors. Firstly, rural areas often lack sufficient healthcare infrastructure, which limits access to preventive measures such as insecticide-treated nets and early treatment options. Environmental conditions in these regions, including more abundant mosquito breeding sites due to stagnant water, agricultural practices, inadequate housing, and limited resources for preventive care, further exacerbate exposure to malaria. This situation is compounded by lower levels of health education, which can delay the recognition of malaria symptoms and hinder timely medical intervention. Collectively, these factors create a heightened vulnerability among young children in rural areas, leading to increased incidences of severe malaria and related complications.

This finding is consistent with previous studies that have identified rural residence as a significant risk factor for malaria complications due to poor sanitation and limited access to healthcare facilities [15]. This suggests that targeted malaria control interventions are needed in rural areas to reduce the risk of malaria complications among children under five in these communities. These interventions may include improving access to effective malaria treatment, increasing the use of insecticide-treated bed nets, and implementing indoor residual spraying programs.

Another important finding was that children under five from households with a lower wealth index were more likely to experience malaria complications. Children from families with a low wealth index in Nigeria face a higher risk of malaria complications compared to their counterparts from families with a high wealth index, primarily due to socioeconomic disparities that affect access to preventive healthcare and living conditions. Families with limited financial resources often cannot afford or access effective malaria prevention tools such as insecticide-treated nets, proper housing, and prompt medical treatment. These conditions are exacerbated in less urbanized areas where healthcare infrastructure is typically underdeveloped, making early diagnosis and treatment of malaria less likely. Additionally, poor nutritional status common among lower wealth groups can weaken a child's immune system, reducing their ability to fight off malaria and increasing the likelihood of developing severe complications. This combination of limited healthcare access, inadequate living conditions, and compromised health status places children from poorer families at a significantly increased risk of suffering from serious malaria-related health issues.

This finding is consistent with findings from previous studies that identified children from poorer households as more likely to live in overcrowded and unhygienic environments, which can significantly increase their risk of exposure to malaria [16-18]. This highlights the need to address socioeconomic disparities in malaria prevention and control efforts. Interventions that target vulnerable populations, such as providing free or subsidized bed nets to low-income households or implementing community-based malaria prevention, are essential.

Furthermore, the study found that the mother's education level was a significant predictor of malaria complications in children under five years of age. Children whose mothers had no education had significantly higher odds of developing malaria complications. Children under five from mothers with low levels of education in Nigeria face higher risks of malaria complications compared to their counterparts from mothers with higher levels of education, largely because maternal education significantly influences health knowledge and practices. Educated mothers are more likely to utilize preventive healthcare services, recognize early symptoms of diseases like malaria, and seek timely medical intervention. In contrast, mothers with lower educational levels might not be as aware of the importance of using mosquito nets, managing the environment to reduce mosquito breeding sites, or the critical timing for accessing antimalarial treatment. This lack of knowledge and awareness can lead to delayed treatment and an increased likelihood of severe malaria complications [19].

Additionally, these issues are often magnified in rural settings, where healthcare resources and access to education are generally more limited than in urban areas. Thus, the intersection of low maternal education and inadequate healthcare infrastructure puts these children at a significantly greater risk of adverse outcomes from malaria [20]. This suggests that improving maternal education could have a positive impact on child health outcomes, including the risk of malaria complications. Interventions that aim to improve maternal education, such as providing access to education and training programs for mothers, may have a positive impact on child health outcomes.

Finally, the study found that gender was also significantly associated with malaria complications, with female children being less likely to experience complications compared to male children. This may be related to sociocultural practices. In certain areas, boys might be more exposed to mosquito habitats due to behavioral patterns or care practices that differ by gender. For example, in some communities, young boys are likely to spend more time outside the house during peak mosquito activity periods. This might contribute to higher incidence rates and severity of malaria in male children, especially in rural communities, highlighting the need for targeted intervention strategies that consider these unique risks. This finding aligns with previous studies that have identified male gender as a significant risk factor for malaria complications due to biological and sociocultural factors [20].

This study underscores the necessity for targeted malaria control interventions that address these unique risk factors. Moreover, the outcomes of this study can provide essential information that can be used to inform public health policies and intervention efforts. This information can be used by the World Health Organization to develop targeted interventions, more efficiently allocate resources, improve malaria preventive and treatment programs, and increase monitoring and evaluation efforts [21].

The strengths of this study include its use of a nationally representative sample of children under five, which enhances the representativeness of the findings. The study also took a comprehensive approach by examining a wide range of factors that could contribute to the risk of malaria complications in children under five, such as the type of place of residence, wealth index, mother’s level of education, and gender. These findings have significant public health implications, highlighting the need for interventions aimed at reducing the risk of malaria complications in children under five in Nigeria.

However, the study has some limitations. It relied on self-reported information, which can be subject to recall bias. The study only included children under five and may not be generalizable to older children or adults. Additionally, the study did not include information on the severity of malaria complications, which may have provided more insight into the impact of the examined variables.

## Conclusions

In conclusion, the findings of this study have important implications for malaria prevention and control efforts in Nigeria. Addressing socioeconomic disparities, improving access to effective malaria treatment, and implementing targeted interventions for vulnerable populations may help reduce the risk of malaria complications in children under five in Nigeria. The next step in this analysis will be to conduct further studies to evaluate the effectiveness of the existing interventions in reducing the burden of malaria complications in children under five in Nigeria. The findings from this project can be used by the WHO and public health authorities to develop targeted interventions to reduce the risk of malaria complications in children under five in Nigeria.

With the knowledge of the significant association between the risk of malaria complications and factors such as type of place of residence, wealth index, mother’s level of education, and gender, public health officials can develop targeted intervention strategies that focus on these variables. For instance, interventions could be designed to improve living conditions in rural areas or to provide more education to mothers in these areas on how to prevent and treat malaria.

The findings from this study can be used to allocate resources more efficiently. Public health officials can prioritize rural areas and direct more resources toward those regions to reduce the risk of the disease. The information from this study can be used to enhance malaria prevention and treatment programs in Nigeria. For instance, public health professionals can develop targeted programs to prevent and treat malaria in rural areas of Nigeria. The study findings can also be used to bolster monitoring and evaluation efforts. Public health officials can use the data to monitor the impact of interventions and evaluate their effectiveness in reducing the risk of malaria complications in children under five in Nigeria.
